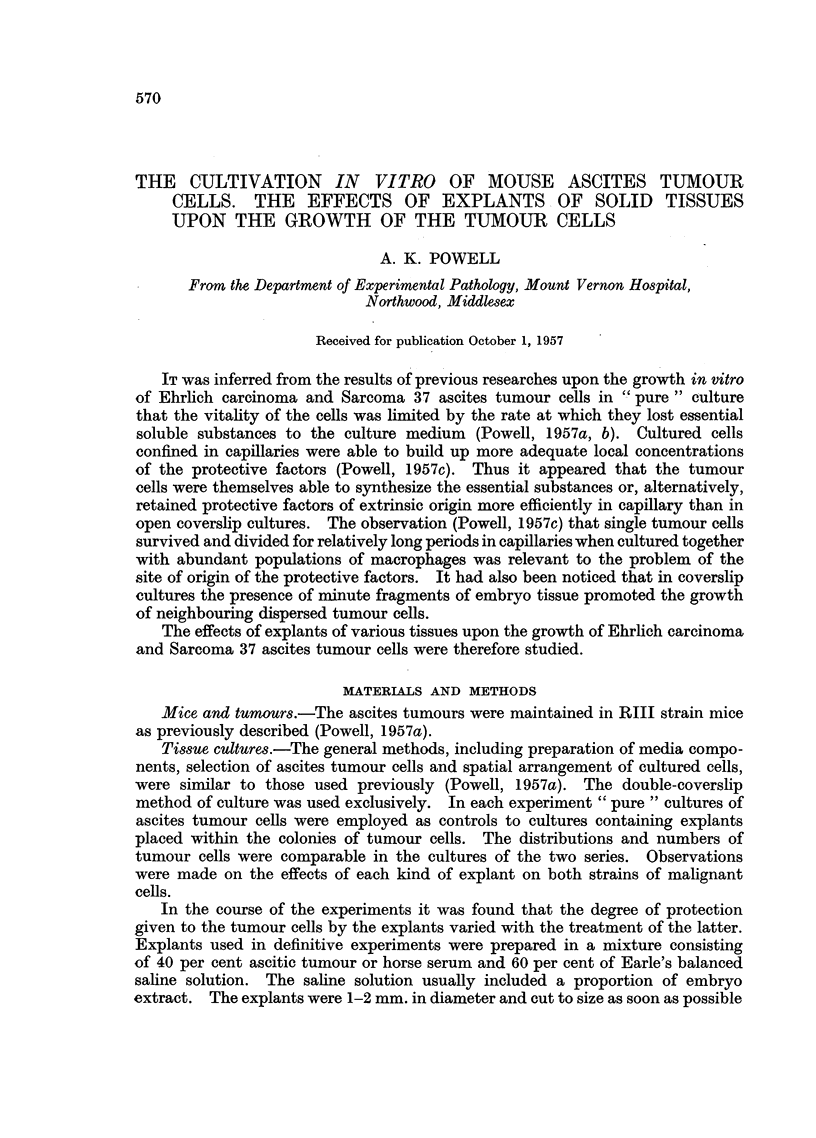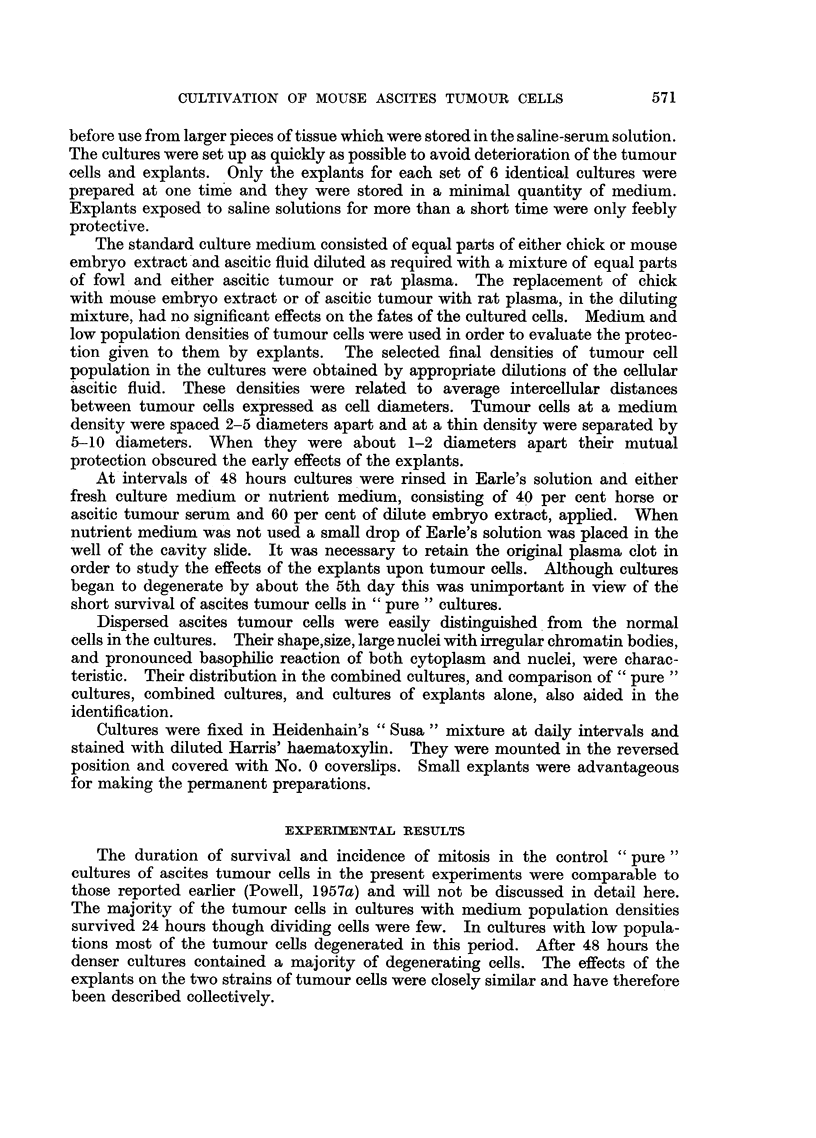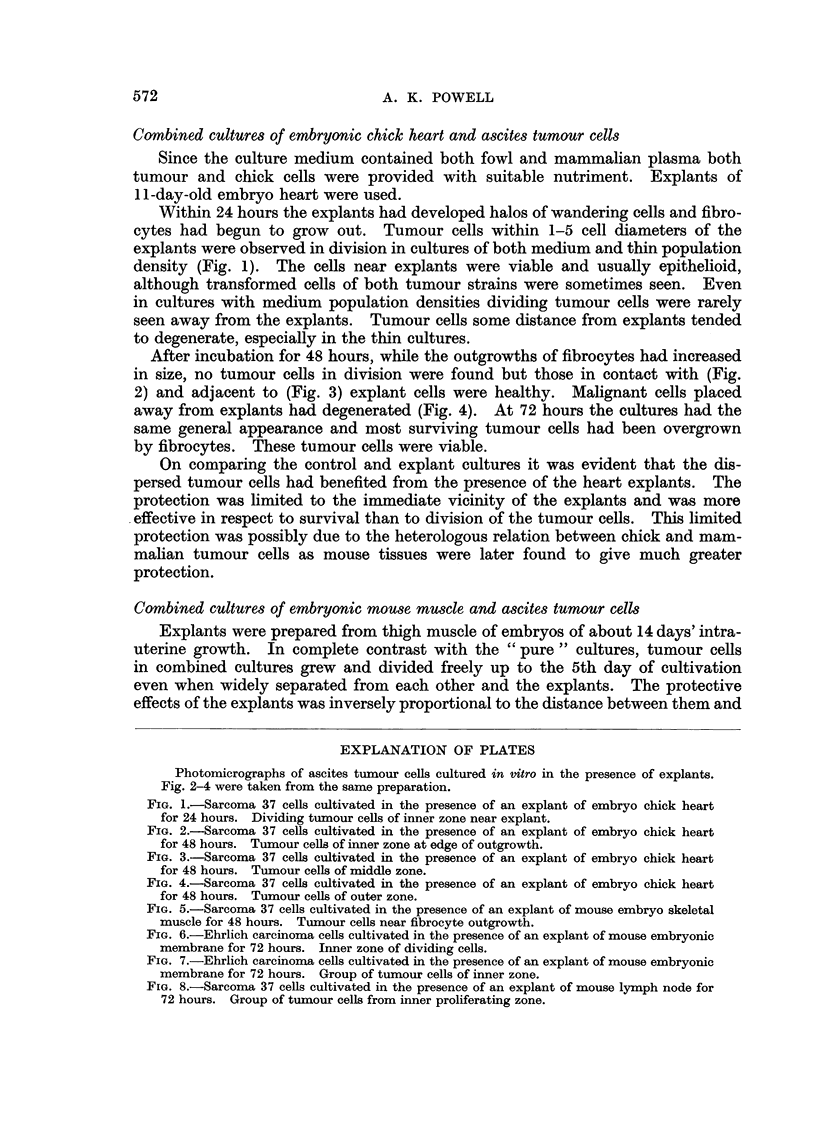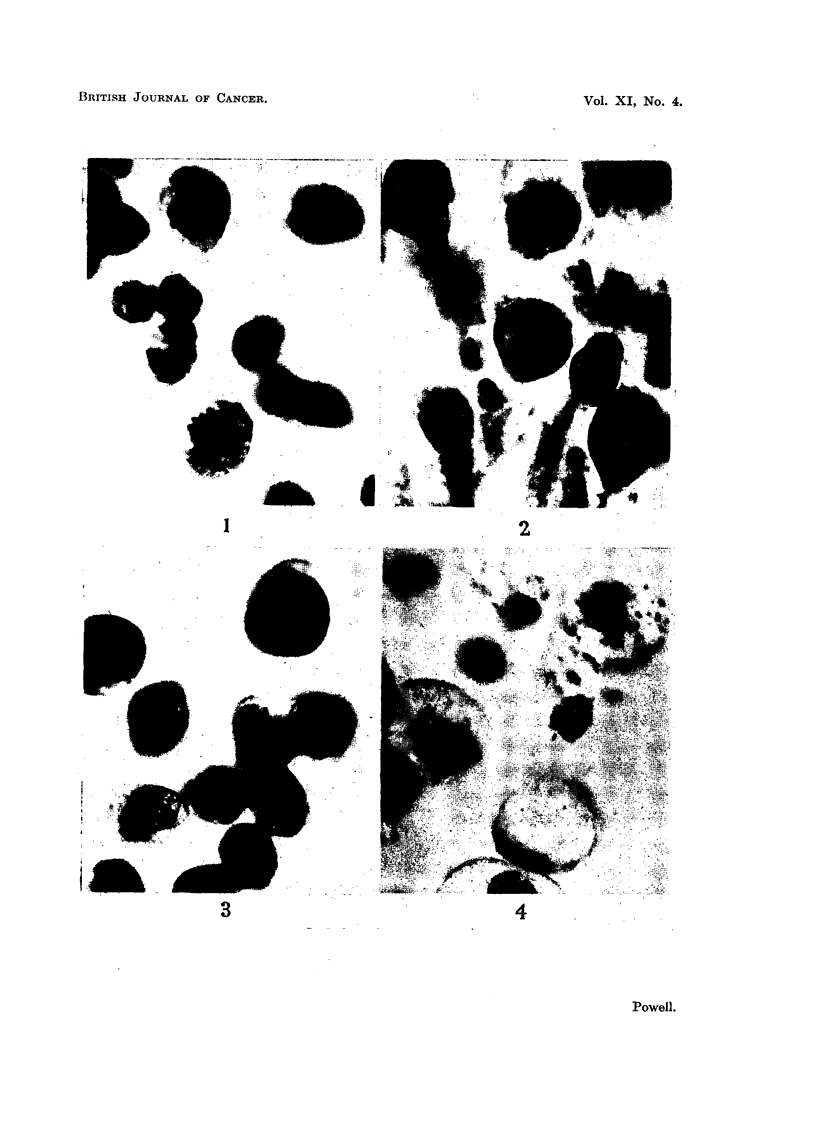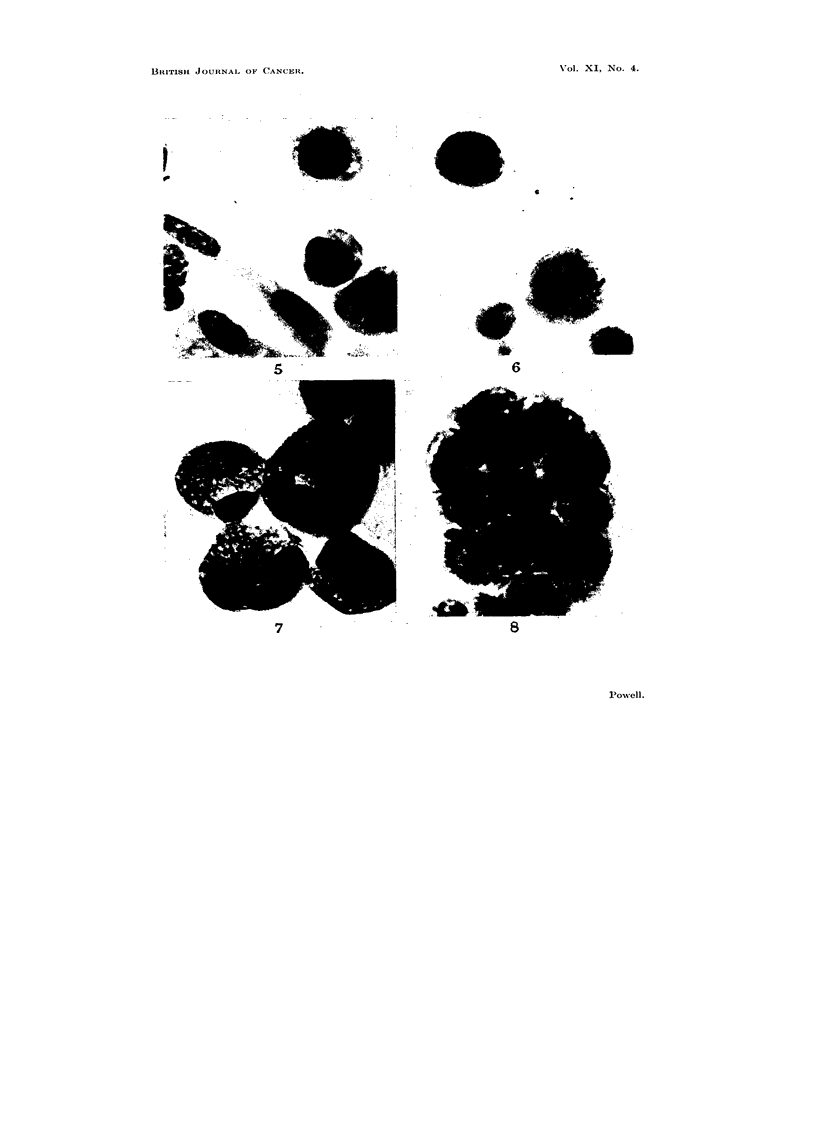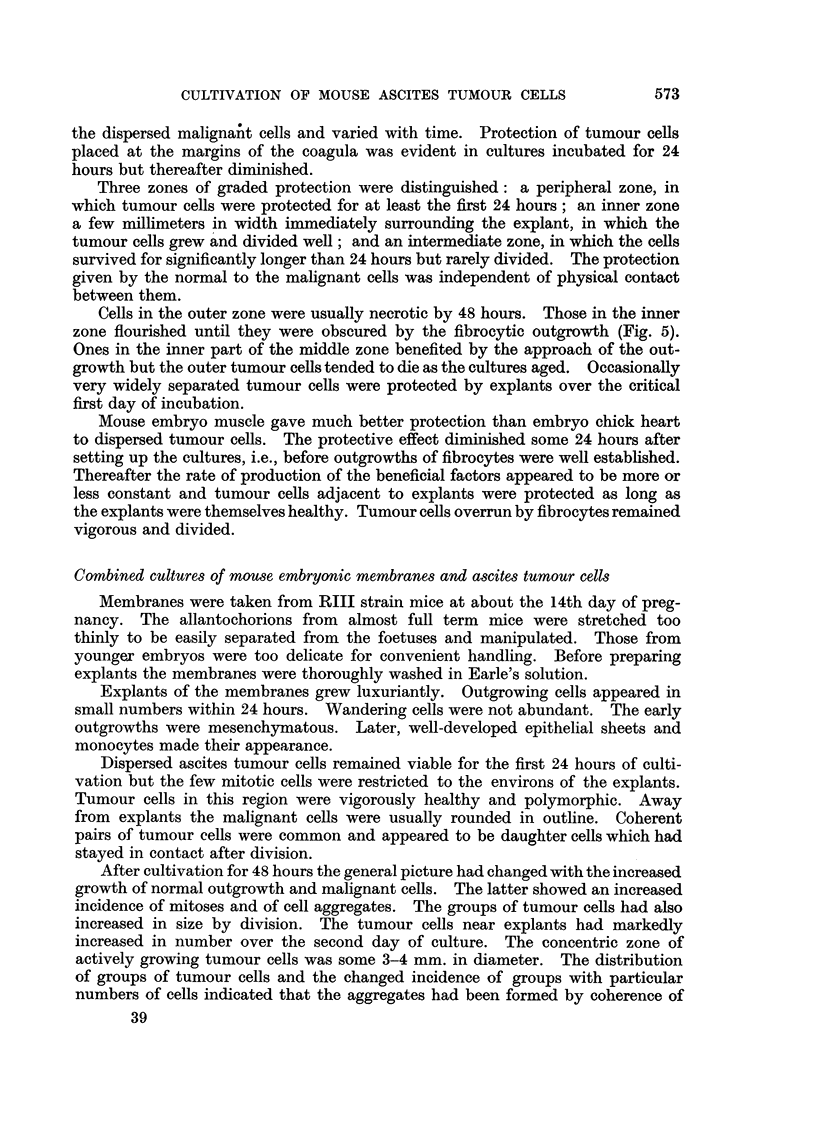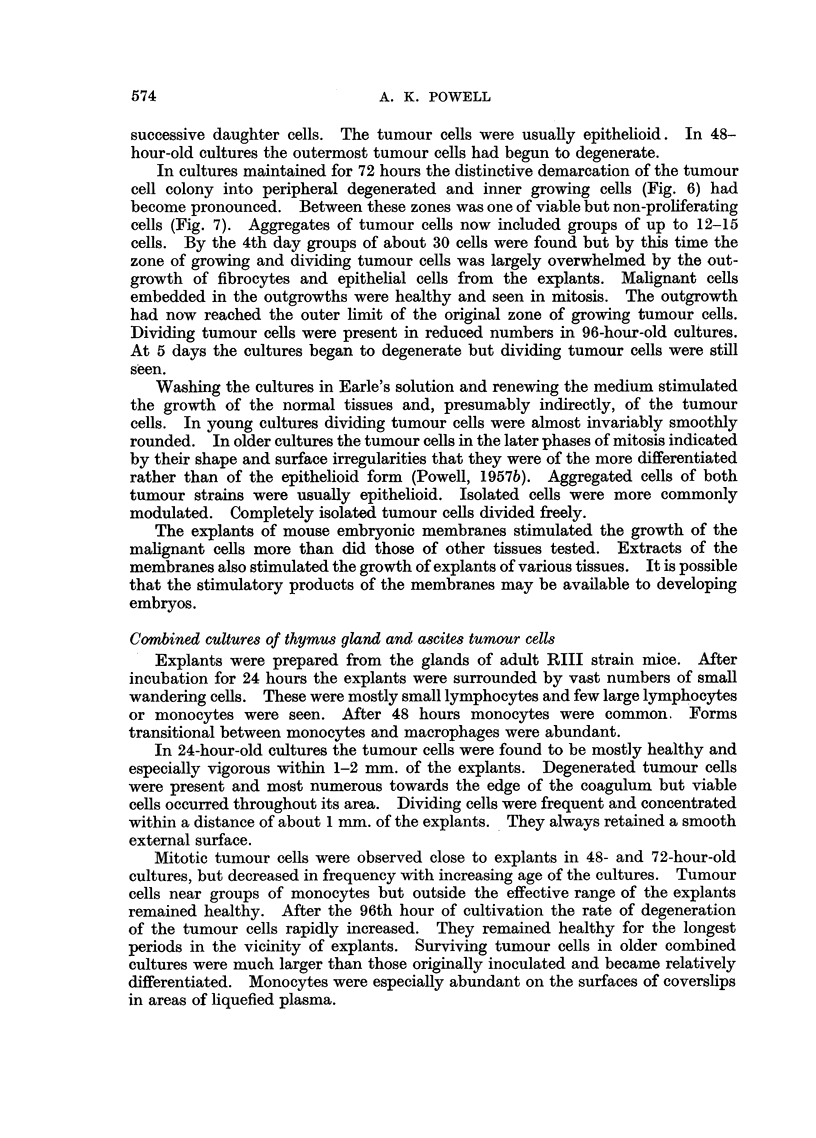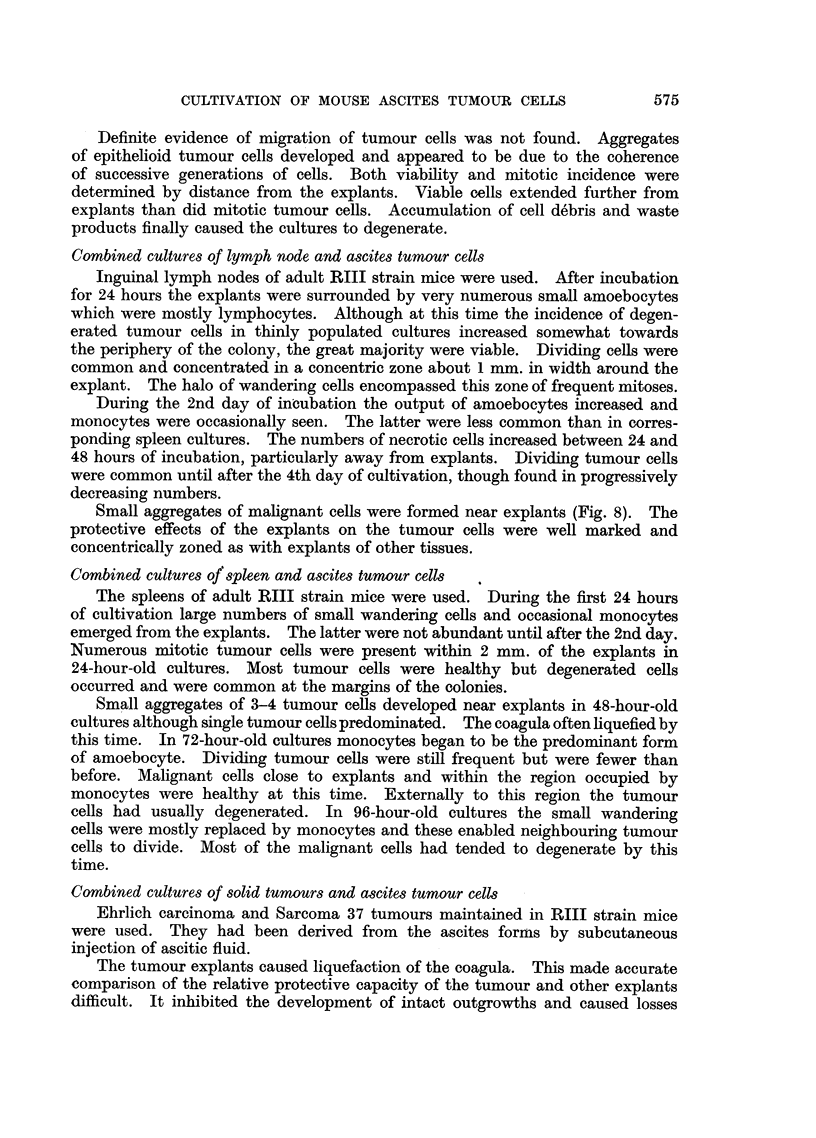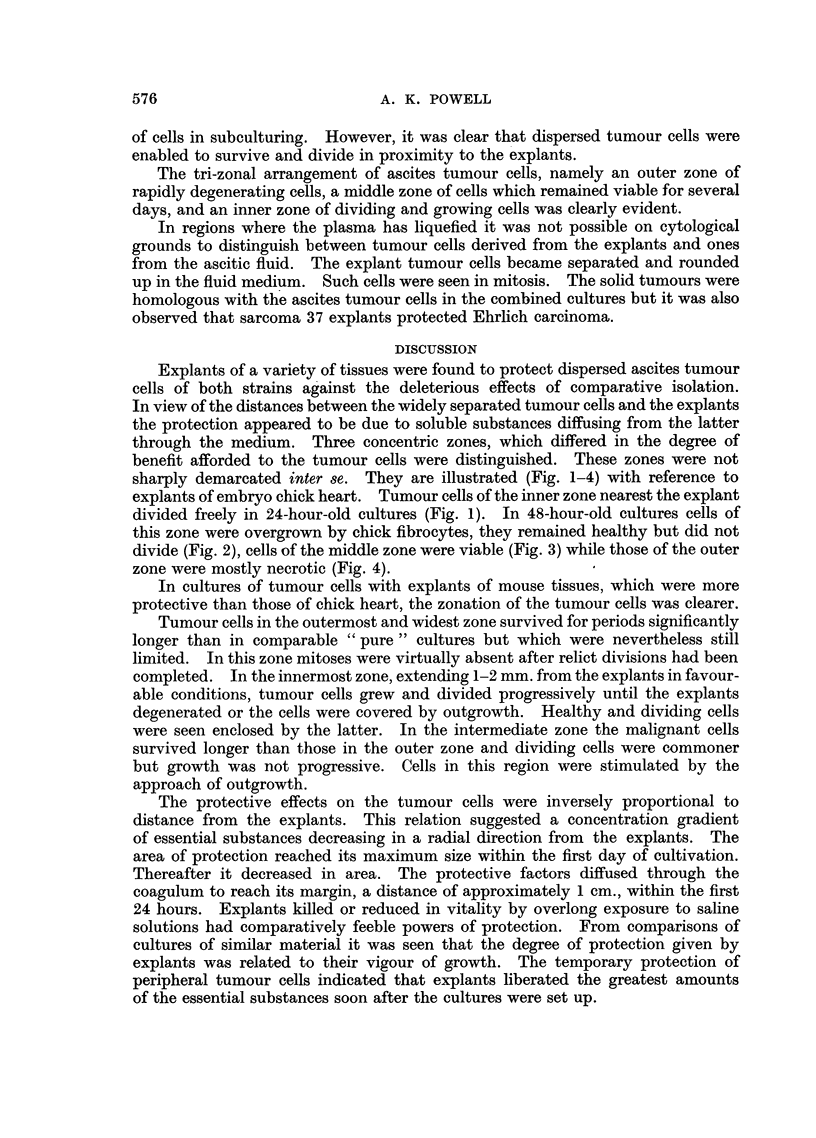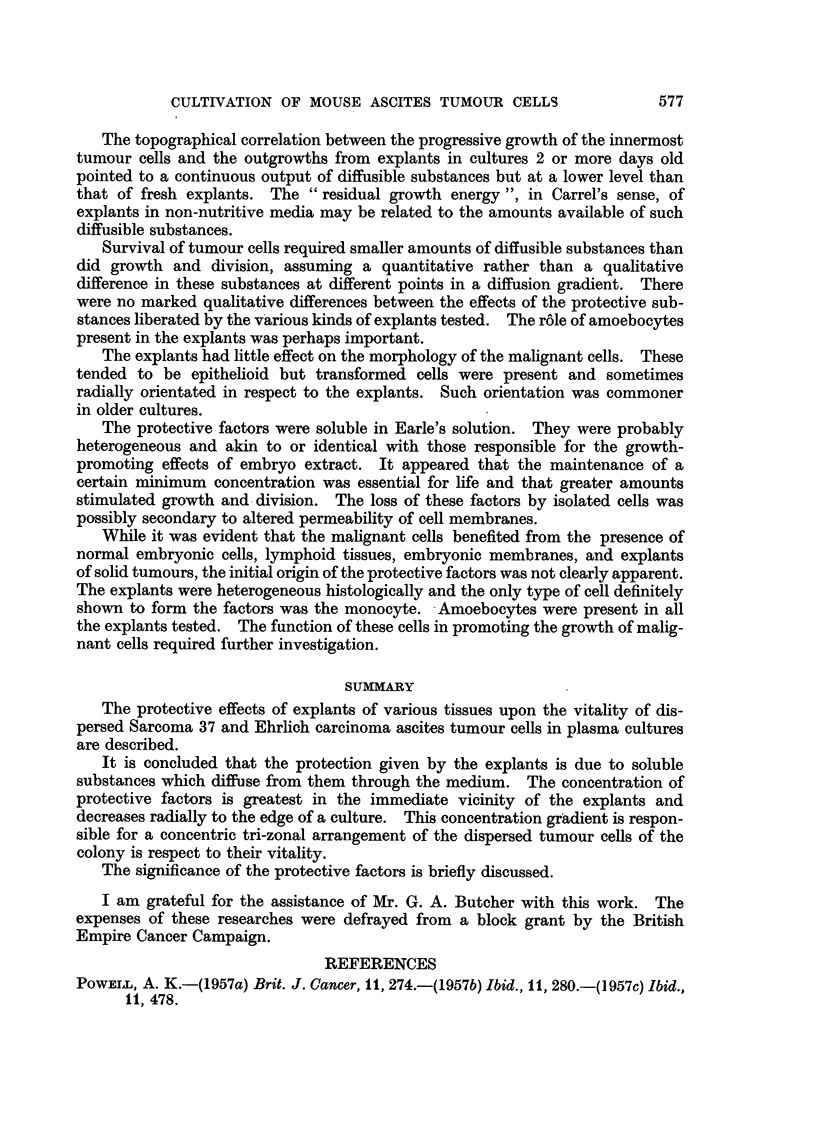# The Cultivation in vitro of Mouse Ascites Tumour Cells. The Effects of Explants of Solid Tissues upon the Growth of the Tumour Cells

**DOI:** 10.1038/bjc.1957.70

**Published:** 1957-12

**Authors:** A. K. Powell

## Abstract

**Images:**


### THE CULTIVATION *IN VITRO* OF MOUSE ASCITES TUMOUR CELLS. THE EFFECTS OF EXPLANTS OF SOLID TISSUES UPON THE GROWTH OF THE TUMOUR CELLS

A. K. POWELL

*From the Department of Experimental Pathology, Mount Vernon Hospital, Northwood, Middlesex*

Received for publication October 1, 1957

IT was inferred from the results of previous researches upon the growth *in vitro* of Ehrlich carcinoma and Sarcoma 37 ascites tumour cells in " pure " culture that the vitality of the cells was limited by the rate at which they lost essential soluble substances to the culture medium (Powell, 1957*a*, *b*). Cultured cells confined in capillaries were able to build up more adequate local concentrations of the protective factors (Powell, 1957*c*). Thus it appeared that the tumour cells were themselves able to synthesize the essential substances or, alternatively, retained protective factors of extrinsic origin more efficiently in capillary than in open coverslip cultures. The observation (Powell, 1957*c*) that single tumour cells survived and divided for relatively long periods in capillaries when cultured together with abundant populations of macrophages was relevant to the problem of the site of origin of the protective factors. It had also been noticed that in coverslip cultures the presence of minute fragments of embryo tissue promoted the growth of neighbouring dispersed tumour cells.

The effects of explants of various tissues upon the growth of Ehrlich carcinoma and Sarcoma 37 ascites tumour cells were therefore studied.

### MATERIALS AND METHODS

*Mice and tumours.*—The ascites tumours were maintained in RIII strain mice as previously described (Powell, 1957*a*).

*Tissue cultures.*—The general methods, including preparation of media components, selection of ascites tumour cells and spatial arrangement of cultured cells, were similar to those used previously (Powell, 1957*a*). The double-coverslip method of culture was used exclusively. In each experiment " pure " cultures of ascites tumour cells were employed as controls to cultures containing explants placed within the colonies of tumour cells. The distributions and numbers of tumour cells were comparable in the cultures of the two series. Observations were made on the effects of each kind of explant on both strains of malignant cells.

In the course of the experiments it was found that the degree of protection given to the tumour cells by the explants varied with the treatment of the latter. Explants used in definitive experiments were prepared in a mixture consisting of 40 per cent ascitic tumour or horse serum and 60 per cent of Earle's balanced saline solution. The saline solution usually included a proportion of embryo extract. The explants were 1–2 mm. in diameter and cut to size as soon as possible before use from larger pieces of tissue which were stored in the saline-serum solution. The cultures were set up as quickly as possible to avoid deterioration of the tumour cells and explants. Only the explants for each set of 6 identical cultures were prepared at one time and they were stored in a minimal quantity of medium. Explants exposed to saline solutions for more than a short time were only feebly protective.

The standard culture medium consisted of equal parts of either chick or mouse embryo extract and ascitic fluid diluted as required with a mixture of equal parts of fowl and either ascitic tumour or rat plasma. The replacement of chick with mouse embryo extract or of ascitic tumour with rat plasma, in the diluting mixture, had no significant effects on the fates of the cultured cells. Medium and low population densities of tumour cells were used in order to evaluate the protection given to them by explants. The selected final densities of tumour cell population in the cultures were obtained by appropriate dilutions of the cellular ascitic fluid. These densities were related to average intercellular distances between tumour cells expressed as cell diameters. Tumour cells at a medium density were spaced 2–5 diameters apart and at a thin density were separated by 5–10 diameters. When they were about 1–2 diameters apart their mutual protection obscured the early effects of the explants.

At intervals of 48 hours cultures were rinsed in Earle's solution and either fresh culture medium or nutrient medium, consisting of 40 per cent horse or ascitic tumour serum and 60 per cent of dilute embryo extract, applied. When nutrient medium was not used a small drop of Earle's solution was placed in the well of the cavity slide. It was necessary to retain the original plasma clot in order to study the effects of the explants upon tumour cells. Although cultures began to degenerate by about the 5th day this was unimportant in view of the short survival of ascites tumour cells in " pure " cultures.

Dispersed ascites tumour cells were easily distinguished from the normal cells in the cultures. Their shape, size, large nuclei with irregular chromatin bodies, and pronounced basophilic reaction of both cytoplasm and nuclei, were characteristic. Their distribution in the combined cultures, and comparison of " pure " cultures, combined cultures, and cultures of explants alone, also aided in the identification.

Cultures were fixed in Heidenhain's " Susa " mixture at daily intervals and stained with diluted Harris' haematoxylin. They were mounted in the reversed position and covered with No. 0 coverslips. Small explants were advantageous for making the permanent preparations.

### EXPERIMENTAL RESULTS

The duration of survival and incidence of mitosis in the control " pure " cultures of ascites tumour cells in the present experiments were comparable to those reported earlier (Powell, 1957*a*) and will not be discussed in detail here. The majority of the tumour cells in cultures with medium population densities survived 24 hours though dividing cells were few. In cultures with low populations most of the tumour cells degenerated in this period. After 48 hours the denser cultures contained a majority of degenerating cells. The effects of the explants on the two strains of tumour cells were closely similar and have therefore been described collectively.

*Combined cultures of embryonic chick heart and ascites tumour cells*

Since the culture medium contained both fowl and mammalian plasma both tumour and chick cells were provided with suitable nutriment. Explants of 11-day-old embryo heart were used.

Within 24 hours the explants had developed halos of wandering cells and fibrocytes had begun to grow out. Tumour cells within 1–5 cell diameters of the explants were observed in division in cultures of both medium and thin population density (Fig. 1). The cells near explants were viable and usually epithelioid, although transformed cells of both tumour strains were sometimes seen. Even in cultures with medium population densities dividing tumour cells were rarely seen away from the explants. Tumour cells some distance from explants tended to degenerate, especially in the thin cultures.

After incubation for 48 hours, while the outgrowths of fibrocytes had increased in size, no tumour cells in division were found but those in contact with (Fig. 2) and adjacent to (Fig. 3) explant cells were healthy. Malignant cells placed away from explants had degenerated (Fig. 4). At 72 hours the cultures had the same general appearance and most surviving tumour cells had been overgrown by fibrocytes. These tumour cells were viable.

On comparing the control and explant cultures it was evident that the dispersed tumour cells had benefited from the presence of the heart explants. The protection was limited to the immediate vicinity of the explants and was more effective in respect to survival than to division of the tumour cells. This limited protection was possibly due to the heterologous relation between chick and mammalian tumour cells as mouse tissues were later found to give much greater protection.

*Combined cultures of embryonic mouse muscle and ascites tumour cells*

Explants were prepared from thigh muscle of embryos of about 14 days' intrauterine growth. In complete contrast with the " pure " cultures, tumour cells in combined cultures grew and divided freely up to the 5th day of cultivation even when widely separated from each other and the explants. The protective effects of the explants was inversely proportional to the distance between them and

---

EXPLANATION OF PLATES

Photomicrographs of ascites tumour cells cultured *in vitro* in the presence of explants. Fig. 2–4 were taken from the same preparation.

FIG. 1.—Sarcoma 37 cells cultivated in the presence of an explant of embryo chick heart for 24 hours. Dividing tumour cells of inner zone near explant.

FIG. 2.—Sarcoma 37 cells cultivated in the presence of an explant of embryo chick heart for 48 hours. Tumour cells of inner zone at edge of outgrowth.

FIG. 3.—Sarcoma 37 cells cultivated in the presence of an explant of embryo chick heart for 48 hours. Tumour cells of middle zone.

FIG. 4.—Sarcoma 37 cells cultivated in the presence of an explant of embryo chick heart for 48 hours. Tumour cells of outer zone.

FIG. 5.—Sarcoma 37 cells cultivated in the presence of an explant of mouse embryo skeletal muscle for 48 hours. Tumour cells near fibrocyte outgrowth.

FIG. 6.—Ehrlich carcinoma cells cultivated in the presence of an explant of mouse embryonic membrane for 72 hours. Inner zone of dividing cells.

FIG. 7.—Ehrlich carcinoma cells cultivated in the presence of an explant of mouse embryonic membrane for 72 hours. Group of tumour cells of inner zone.

FIG. 8.—Sarcoma 37 cells cultivated in the presence of an explant of mouse lymph node for 72 hours. Group of tumour cells from inner proliferating zone.

1 2 3 4

Powell.

5 6 7 8

Powell.

the dispersed malignant cells and varied with time. Protection of tumour cells placed at the margins of the coagula was evident in cultures incubated for 24 hours but thereafter diminished.

Three zones of graded protection were distinguished: a peripheral zone, in which tumour cells were protected for at least the first 24 hours; an inner zone a few millimeters in width immediately surrounding the explant, in which the tumour cells grew and divided well; and an intermediate zone, in which the cells survived for significantly longer than 24 hours but rarely divided. The protection given by the normal to the malignant cells was independent of physical contact between them.

Cells in the outer zone were usually necrotic by 48 hours. Those in the inner zone flourished until they were obscured by the fibrocytic outgrowth (Fig. 5). Ones in the inner part of the middle zone benefited by the approach of the outgrowth but the outer tumour cells tended to die as the cultures aged. Occasionally very widely separated tumour cells were protected by explants over the critical first day of incubation.

Mouse embryo muscle gave much better protection than embryo chick heart to dispersed tumour cells. The protective effect diminished some 24 hours after setting up the cultures, i.e., before outgrowths of fibrocytes were well established. Thereafter the rate of production of the beneficial factors appeared to be more or less constant and tumour cells adjacent to explants were protected as long as the explants were themselves healthy. Tumour cells overrun by fibrocytes remained vigorous and divided.

*Combined cultures of mouse embryonic membranes and ascites tumour cells*

Membranes were taken from RIII strain mice at about the 14th day of pregnancy. The allantochorions from almost full term mice were stretched too thinly to be easily separated from the foetuses and manipulated. Those from younger embryos were too delicate for convenient handling. Before preparing explants the membranes were thoroughly washed in Earle's solution.

Explants of the membranes grew luxuriantly. Outgrowing cells appeared in small numbers within 24 hours. Wandering cells were not abundant. The early outgrowths were mesenchymatous. Later, well-developed epithelial sheets and monocytes made their appearance.

Dispersed ascites tumour cells remained viable for the first 24 hours of cultivation but the few mitotic cells were restricted to the environs of the explants. Tumour cells in this region were vigorously healthy and polymorphic. Away from explants the malignant cells were usually rounded in outline. Coherent pairs of tumour cells were common and appeared to be daughter cells which had stayed in contact after division.

After cultivation for 48 hours the general picture had changed with the increased growth of normal outgrowth and malignant cells. The latter showed an increased incidence of mitoses and of cell aggregates. The groups of tumour cells had also increased in size by division. The tumour cells near explants had markedly increased in number over the second day of culture. The concentric zone of actively growing tumour cells was some 3–4 mm. in diameter. The distribution of groups of tumour cells and the changed incidence of groups with particular numbers of cells indicated that the aggregates had been formed by coherence of successive daughter cells. The tumour cells were usually epithelioid. In 48-hour-old cultures the outermost tumour cells had begun to degenerate.

In cultures maintained for 72 hours the distinctive demarcation of the tumour cell colony into peripheral degenerated and inner growing cells (Fig. 6) had become pronounced. Between these zones was one of viable but non-proliferating cells (Fig. 7). Aggregates of tumour cells now included groups of up to 12–15 cells. By the 4th day groups of about 30 cells were found but by this time the zone of growing and dividing tumour cells was largely overwhelmed by the outgrowth of fibrocytes and epithelial cells from the explants. Malignant cells embedded in the outgrowths were healthy and seen in mitosis. The outgrowth had now reached the outer limit of the original zone of growing tumour cells. Dividing tumour cells were present in reduced numbers in 96-hour-old cultures. At 5 days the cultures began to degenerate but dividing tumour cells were still seen.

Washing the cultures in Earle's solution and renewing the medium stimulated the growth of the normal tissues and, presumably indirectly, of the tumour cells. In young cultures dividing tumour cells were almost invariably smoothly rounded. In older cultures the tumour cells in the later phases of mitosis indicated by their shape and surface irregularities that they were of the more differentiated rather than of the epithelioid form (Powell, 1957*b*). Aggregated cells of both tumour strains were usually epithelioid. Isolated cells were more commonly modulated. Completely isolated tumour cells divided freely.

The explants of mouse embryonic membranes stimulated the growth of the malignant cells more than did those of other tissues tested. Extracts of the membranes also stimulated the growth of explants of various tissues. It is possible that the stimulatory products of the membranes may be available to developing embryos.

*Combined cultures of thymus gland and ascites tumour cells*

Explants were prepared from the glands of adult RIII strain mice. After incubation for 24 hours the explants were surrounded by vast numbers of small wandering cells. These were mostly small lymphocytes and few large lymphocytes or monocytes were seen. After 48 hours monocytes were common. Forms transitional between monocytes and macrophages were abundant.

In 24-hour-old cultures the tumour cells were found to be mostly healthy and especially vigorous within 1–2 mm. of the explants. Degenerated tumour cells were present and most numerous towards the edge of the coagulum but viable cells occurred throughout its area. Dividing cells were frequent and concentrated within a distance of about 1 mm. of the explants. They always retained a smooth external surface.

Mitotic tumour cells were observed close to explants in 48- and 72-hour-old cultures, but decreased in frequency with increasing age of the cultures. Tumour cells near groups of monocytes but outside the effective range of the explants remained healthy. After the 96th hour of cultivation the rate of degeneration of the tumour cells rapidly increased. They remained healthy for the longest periods in the vicinity of explants. Surviving tumour cells in older combined cultures were much larger than those originally inoculated and became relatively differentiated. Monocytes were especially abundant on the surfaces of coverslips in areas of liquefied plasma.

Definite evidence of migration of tumour cells was not found. Aggregates of epithelioid tumour cells developed and appeared to be due to the coherence of successive generations of cells. Both viability and mitotic incidence were determined by distance from the explants. Viable cells extended further from explants than did mitotic tumour cells. Accumulation of cell débris and waste products finally caused the cultures to degenerate.

*Combined cultures of lymph node and ascites tumour cells*

Inguinal lymph nodes of adult RIII strain mice were used. After incubation for 24 hours the explants were surrounded by very numerous small amoebocytes which were mostly lymphocytes. Although at this time the incidence of degenerated tumour cells in thinly populated cultures increased somewhat towards the periphery of the colony, the great majority were viable. Dividing cells were common and concentrated in a concentric zone about 1 mm. in width around the explant. The halo of wandering cells encompassed this zone of frequent mitoses.

During the 2nd day of incubation the output of amoebocytes increased and monocytes were occasionally seen. The latter were less common than in corresponding spleen cultures. The numbers of necrotic cells increased between 24 and 48 hours of incubation, particularly away from explants. Dividing tumour cells were common until after the 4th day of cultivation, though found in progressively decreasing numbers.

Small aggregates of malignant cells were formed near explants (Fig. 8). The protective effects of the explants on the tumour cells were well marked and concentrically zoned as with explants of other tissues.

*Combined cultures of spleen and ascites tumour cells*

The spleens of adult RIII strain mice were used. During the first 24 hours of cultivation large numbers of small wandering cells and occasional monocytes emerged from the explants. The latter were not abundant until after the 2nd day. Numerous mitotic tumour cells were present within 2 mm. of the explants in 24-hour-old cultures. Most tumour cells were healthy but degenerated cells occurred and were common at the margins of the colonies.

Small aggregates of 3–4 tumour cells developed near explants in 48-hour-old cultures although single tumour cells predominated. The coagula often liquefied by this time. In 72-hour-old cultures monocytes began to be the predominant form of amoebocyte. Dividing tumour cells were still frequent but were fewer than before. Malignant cells close to explants and within the region occupied by monocytes were healthy at this time. Externally to this region the tumour cells had usually degenerated. In 96-hour-old cultures the small wandering cells were mostly replaced by monocytes and these enabled neighbouring tumour cells to divide. Most of the malignant cells had tended to degenerate by this time.

*Combined cultures of solid tumours and ascites tumour cells*

Ehrlich carcinoma and Sarcoma 37 tumours maintained in RIII strain mice were used. They had been derived from the ascites forms by subcutaneous injection of ascitic fluid.

The tumour explants caused liquefaction of the coagula. This made accurate comparison of the relative protective capacity of the tumour and other explants difficult. It inhibited the development of intact outgrowths and caused losses of cells in subculturing. However, it was clear that dispersed tumour cells were enabled to survive and divide in proximity to the explants.

The tri-zonal arrangement of ascites tumour cells, namely an outer zone of rapidly degenerating cells, a middle zone of cells which remained viable for several days, and an inner zone of dividing and growing cells was clearly evident.

In regions where the plasma has liquefied it was not possible on cytological grounds to distinguish between tumour cells derived from the explants and ones from the ascitic fluid. The explant tumour cells became separated and rounded up in the fluid medium. Such cells were seen in mitosis. The solid tumours were homologous with the ascites tumour cells in the combined cultures but it was also observed that sarcoma 37 explants protected Ehrlich carcinoma.

### DISCUSSION

Explants of a variety of tissues were found to protect dispersed ascites tumour cells of both strains against the deleterious effects of comparative isolation. In view of the distances between the widely separated tumour cells and the explants the protection appeared to be due to soluble substances diffusing from the latter through the medium. Three concentric zones, which differed in the degree of benefit afforded to the tumour cells were distinguished. These zones were not sharply demarcated *inter se*. They are illustrated (Fig. 1–4) with reference to explants of embryo chick heart. Tumour cells of the inner zone nearest the explant divided freely in 24-hour-old cultures (Fig. 1). In 48-hour-old cultures cells of this zone were overgrown by chick fibrocytes, they remained healthy but did not divide (Fig. 2), cells of the middle zone were viable (Fig. 3) while those of the outer zone were mostly necrotic (Fig. 4).

In cultures of tumour cells with explants of mouse tissues, which were more protective than those of chick heart, the zonation of the tumour cells was clearer.

Tumour cells in the outermost and widest zone survived for periods significantly longer than in comparable " pure " cultures but which were nevertheless still limited. In this zone mitoses were virtually absent after relict divisions had been completed. In the innermost zone, extending 1–2 mm. from the explants in favourable conditions, tumour cells grew and divided progressively until the explants degenerated or the cells were covered by outgrowth. Healthy and dividing cells were seen enclosed by the latter. In the intermediate zone the malignant cells survived longer than those in the outer zone and dividing cells were commoner but growth was not progressive. Cells in this region were stimulated by the approach of outgrowth.

The protective effects on the tumour cells were inversely proportional to distance from the explants. This relation suggested a concentration gradient of essential substances decreasing in a radial direction from the explants. The area of protection reached its maximum size within the first day of cultivation. Thereafter it decreased in area. The protective factors diffused through the coagulum to reach its margin, a distance of approximately 1 cm., within the first 24 hours. Explants killed or reduced in vitality by overlong exposure to saline solutions had comparatively feeble powers of protection. From comparisons of cultures of similar material it was seen that the degree of protection given by explants was related to their vigour of growth. The temporary protection of peripheral tumour cells indicated that explants liberated the greatest amounts of the essential substances soon after the cultures were set up.

The topographical correlation between the progressive growth of the innermost tumour cells and the outgrowths from explants in cultures 2 or more days old pointed to a continuous output of diffusible substances but at a lower level than that of fresh explants. The " residual growth energy ", in Carrel's sense, of explants in non-nutritive media may be related to the amounts available of such diffusible substances.

Survival of tumour cells required smaller amounts of diffusible substances than did growth and division, assuming a quantitative rather than a qualitative difference in these substances at different points in a diffusion gradient. There were no marked qualitative differences between the effects of the protective substances liberated by the various kinds of explants tested. The rôle of amoebocytes present in the explants was perhaps important.

The explants had little effect on the morphology of the malignant cells. These tended to be epithelioid but transformed cells were present and sometimes radially orientated in respect to the explants. Such orientation was commoner in older cultures.

The protective factors were soluble in Earle's solution. They were probably heterogeneous and akin to or identical with those responsible for the growth-promoting effects of embryo extract. It appeared that the maintenance of a certain minimum concentration was essential for life and that greater amounts stimulated growth and division. The loss of these factors by isolated cells was possibly secondary to altered permeability of cell membranes.

While it was evident that the malignant cells benefited from the presence of normal embryonic cells, lymphoid tissues, embryonic membranes, and explants of solid tumours, the initial origin of the protective factors was not clearly apparent. The explants were heterogeneous histologically and the only type of cell definitely shown to form the factors was the monocyte. Amoebocytes were present in all the explants tested. The function of these cells in promoting the growth of malignant cells required further investigation.

### SUMMARY

The protective effects of explants of various tissues upon the vitality of dispersed Sarcoma 37 and Ehrlich carcinoma ascites tumour cells in plasma cultures are described.

It is concluded that the protection given by the explants is due to soluble substances which diffuse from them through the medium. The concentration of protective factors is greatest in the immediate vicinity of the explants and decreases radially to the edge of a culture. This concentration gradient is responsible for a concentric tri-zonal arrangement of the dispersed tumour cells of the colony is respect to their vitality.

The significance of the protective factors is briefly discussed.

I am grateful for the assistance of Mr. G. A. Butcher with this work. The expenses of these researches were defrayed from a block grant by the British Empire Cancer Campaign.

### REFERENCES

POWELL, A. K.—(1957*a*) *Brit. J. Cancer*, **11**, 274.—(1957*b*) *Ibid.*, **11**, 280.—(1957*c*) *Ibid.*, **11**, 478.